# Calcium and TRPV4 promote metastasis by regulating cytoskeleton through the RhoA/ROCK1 pathway in endometrial cancer

**DOI:** 10.1038/s41419-020-03181-7

**Published:** 2020-11-23

**Authors:** Xingchen Li, Yuan Cheng, Zhiqi Wang, Jingyi Zhou, Yuanyuan Jia, Xiangjun He, Lijun Zhao, Yangyang Dong, Yuan Fan, Xiao Yang, Boqiang Shen, Xiaotong Wu, Jiaqi Wang, Chunyang Xiong, Lihui Wei, Xiaoping Li, Jianliu Wang

**Affiliations:** 1 Department of Obstetrics and Gynecology, Peking University People’s Hospital, Beijing, 100044 China; 2 Beijing Key Laboratory of Female Pelvic Floor Disorders Diseases, Beijing, 100044 China; 3 Department of Central Laboratory & Institute of Clinical Molecular Biology, Peking University People’s Hospital, Beijing, 100044 China; 4 Department of Mechanics and Bioengineering, College of Engineering, Peking University, Beijing, 100871 China

**Keywords:** Metastasis, Proteomics

## Abstract

Transient receptor potential vanilloid 4 (TRPV4) is a calcium-permeable cation channel that has been associated with several types of cancer. However, its biological significance, as well as its related mechanism in endometrial cancer (EC) still remains elusive. In this study, we examined the function of calcium in EC, with a specific focus on TRPV4 and its downstream pathway. We reported here on the findings that a high level of serum ionized calcium was significantly correlated with advanced EC progression, and among all the calcium channels, TRPV4 played an essential role, with high levels of TRPV4 expression associated with cancer progression both in vitro and in vivo. Proteomic and bioinformatics analysis revealed that TRPV4 was involved in cytoskeleton regulation and Rho protein pathway, which regulated EC cell migration. Mechanistic investigation demonstrated that TRPV4 and calcium influx acted on the cytoskeleton via the RhoA/ROCK1 pathway, ending with LIMK/cofilin activation, which had an impact on F-actin and paxillin (PXN) levels. Overall, our findings indicated that ionized serum calcium level was significantly associated with poor outcomes and calcium channel TRPV4 should be targeted to improve therapeutic and preventive strategies in EC.

## Background

Endometrial carcinoma (EC) is a significant public health burden and a very serious disease for the individual patient. In addition, the therapy of advanced and metastatic cases is not sufficiently efficacious, especially in developed countries^1^, with the incidence of EC reaching 63.4 per 100 thousand in China in 2015^2^. While the overall 5-year survival rate ranges from 74% to 91% in the early stages, the survival of stage IV EC is dramatically lower, at 20–26%. Distant metastasis is the most common cause of lethality in EC^3^. Therefore, research on the cell biological and molecular mechanisms regarding to EC migration, an important mechanism involved in metastasis formation, is warranted.

Our previous work demonstrated that increased ionized calcium promoted the metastasis of peritoneal tumor cells and lymph node metastasis in EC^4,5^, a finding in keeping with other evidence that cytosolic free calcium concentration played an important role in the regulation of downstream cellular functions in cancer^6^. The elevation of calcium concentration was commonly seen in paraneoplastic or bone-metastatic tumors, but seldom in other tumors. Calcium concentration was tightly controlled by calcium channels^7^, and different cancers were linked to the expression of different calcium-related channels, such as TRPM7 in breast cancer^8^, Orai3 in breast cancer^9^, and BKCa in EC^10^. In gastric cancer, calcium enhanced the expression of calcium-sensitive receptor (CaSR) and targeted TRPV4 to promote metastasis^11^. Our previous research found the L-type calcium channel Cav1.3 was required for estrogen-stimulated calcium influx and contributed to the proliferation and migration of EC cells^12^.

The current study focused on the calcium channel TRPV4, a type of transient receptor potential (TRP) family and played a critical role in cell migration^13^. TRP channels constituted a superfamily of polymodal non-selective cation channels and could be activated by a variety of stimuli including ligands, temperature, pH changes, tension, and pressure^14^. TRPV4 was a member of the vanilloid subfamily that regulated calcium homeostasis, and acted to exert an impact on cell adhesion and migration by promoting changes in actin cytoskeleton^15^. Its effect on actin made TRPV4 an important candidate for influencing metastasis in EC, as cell migration required cytoskeletal reorganization^16^: metastasis in cancer involved in a series of aberrant expressions of cytoskeletal proteins, which ultimately lead to the reorganization of actin and migration of cancer cells^17^. Previous research suggested that TRPV4 mediated cancer cell stiffness through controlling the actin cortex^18^. However, the bio-functional role of TRPV4 in cancer progression had not been examined in EC, and little was known of the specific mechanisms by which TRPV4 might act.

One of the most common mechanisms for regulating actin was via the Rho family GTPase pathway, which could be triggered by calcium influx and affected the local assembly or disassembly of F-actin directly and indirectly^19^. In the current study, we tested for evidence that TRPV4-mediated calcium influx lead to actin reorganization and cell migration via the pathway triggered by RhoA. RhoA was a small GTPase that interacted with a downstream effector, Rho-associated protein kinase (ROCK). Previous research suggested that the downstream effector proteins of RhoA mediated tumor cell migration, invasion, and metastasis through changes in the cytoskeleton^17^.

The current study employed a range of in vitro and in vivo methods to establish links among TRPV4, calcium influx, EC prognosis, and cell migration for the first time. We also confirmed that TRPV4-induced calcium changes ultimately affected cytoskeletal actin via the RhoA and its downstream pathway.

## Materials and methods

### Patients and data collection for retrospective clinical correlation

The retrospective clinical correlation analysis was conducted with EC patients who were diagnosed with EC and had undergone surgical treatment. The demographic and clinicopathological parameters including age and BMI were collected from the electronic medical database of the Peking University People’s Hospital (Beijing, China) between January 2012 and December 2016. For analyses comparing preoperative serum calcium levels in tumors with different metastatic ability, we collected postoperative clinical features including FIGO stage, tumor grade (G), lymph node metastasis (LNM), ascites cytology, and lymph-vascular space invasion (LVSI). This study was approved by the Institutional Review Boards of Peking University People’s Hospital (2015PHB116-01). The biologically active fraction of serum calcium was calculated by the following formula: Ionized calcium (mM) = total calcium (mM) + 0.8*(4-Albumin (g/dL).

### Bioinformatics Analysis of TRPV4 in EC

The NCBI Gene Expression Omnibus (GEO, GSE17025) and The Cancer Genome Atlas (TCGA) datasets containing human EC gene expression profiles were used in analyses relating TRPV4 expression to clinicopathological factors, and identifying the functions regulated by TRPV4. In total, 491 primary EC patients and 23 normal endometrial tissue with detailed TRPV4 expression and complete clinical data were chosen from the database in TCGA. In GEO database, 91 EC patients and 12 normal tissues were retrieved. “*DESeq*” and “*limma*” packages of R software were used to identify differentially expressed genes (DEGs) between normal endometrial tissues and EC tissues. The R package “*ggplot2*” was used to draw the boxplots to visualize expression differences for discrete variables. TRPV4 expression level was retrieved, and high- and low-expression conditions assigned according to median value of TRPV4 expression. GSEA 3.0 was used to identify enriched functions and pathways in high- and low-expression of TRPV4 groups.

### Calcium channel selection and heatmap

We searched for gene names containing “calcium channel” in PubMed and downloaded the gene list (Fig. 2a). “Org name” of “homo sapiens” were left and other types were deleted. Afterward, 23 EC tissues and the corresponding normal pairs were compared in order to create a differential gene set with logFC (fold change) greater than 2. A Venn diagram was used to identify the common and unique genes, which implicated the calcium channel that played an important role in EC. A heatmap was plotted by “*pheatmap*” R package (version 0.7.7).

### Antibodies and reagents

TRPV4, ROCK1, ROCK2, and paxillin (PXN) antibodies were purchased from Abcam (Cambridge, MA, USA). RhoA, RhoB, RhoC, CDC42, RAC1/2, LIMK, phospho-LIMK, cofilin, phospho-cofilin, MLC, phospho-MLC, MYPT, and phospho-MYPT antibodies were purchased from Cell Signaling Technology (Beverly, MA, USA). GAPDH were purchased from Proteintech (Rosemont, IL, USA). Rho pathway antagonist Y27632, calcium chelator BAPTA-AM, TRPV4 agonist GSK1016790A, and its antagonist HC067047 were purchased from Selleck (Shanghai, China). Opti-MEM medium and Lipofectamine RNAiMAX reagent, Fluo-4 AM, Rhodamine Phalloidin, puromycin, and DAPI were purchased from Invitrogen (Massachusetts, USA).

### Immunohistochemistry (IHC)

In total 15 resected normal endometrial tissues and 15 EC tissues were obtained for immunohistochemistry. Resection of all the tissues was approved by the Institutional Review Boards of Peking University People’s Hospital. Complete demographic information was collected from the electronic record. The diagnosis was confirmed pathologically. The immunohistological analysis for TRPV4 in EC and normal endometrial tissue was performed as previously described^20^. The Image-pro Plus 6.0 was used to assess the expression level of TRPV4.

### Cell culture

The EC cell lines HEC-1A, HEC-1B, ishikawa, AN3CA, KLE, and RL9-52 were obtained from laboratory stocks in Peking University People’s Hospital and cultured at 5% CO2 and 37 °C in DMEM/F12, Mcroy5A and DMEM medium (Gibco, Invitrogen, Carlsbad, CA, USA) supplied with 10% fetal bovine serum (Gibco, Invitrogen). Cells were subcultured every 2–3 days, and used for cell-based experiments (e.g., gap closure, transwell, and Western blot analyses).

### Cell transfections of recombinant lentivirus

Lentivirus (LV) construction targeting depletion or overexpression of TRPV4, and the corresponding negative controls (NC) were obtained from the Genechem Company (Shanghai, China). The LV vector containing the GFP gene was used to verify transfection efficiency. Recombinant viruses were collected and purified, and titer was determined following the manufacturer’s instructions. For transfecting ishikawa cell line, cells (5 × 10^4^) were seeded into a 24-well culture plate. Sixteen hours later, ishikawa cells were transfected with LV-shNC or LV-shTRPV4. After 24 h transfection, green fluorescence was captured by fluorescence microscope (Leica DMI 6000B) and then verified by Western blotting. Stable transfected cell lines were obtained with 4 μg/mL puromycin selection for 48 h and were maintained in medium containing 2 μg/mL puromycin for 2 weeks. For the transfection of the HEC-1A cell line, LV-OE-NC or LV-OE-TRPV4 was cultured together with the cells for 24 h, and then puromycin was used for selection.

### siRNA Plasmid Transfections

The short interfering RNA (siRNA) overexpressing RhoA and respective negative plasmid siRNA were chemically synthesized by Gene Pharma (Suzhou, China). Plasmid transfections were conducted using Opti-MEM medium and Lipofectamine RNAiMAX reagent according to the manufacturer’s instructions.

### Transwell migration assay

Chamber dishes (NEST) were prepared as previously described^21^. EC cells of different groups at a density of 1 × 10^5^ cells per well were added into the upper chamber and incubated for 24 h. Then cells were fixed with 4% paraformaldehyde for 30 minutes, stained with 0.1% crystal violet for 5 minutes, washed 3 times with PBS, and counted in 6 fields under the microscope. All experiments were repeated 3 times.

### Gap closure assay

In order to evaluate the influence of TRPV4 on ishikawa and HEC-1A cell mobility, different confluent EC cells in a 6-well plate were scratched carefully using 200 μl sterile pipette tips. After scratching, cell debris was washed away with PBS. Images were taken at 0 and 24 h and analyzed using Image J software (Rawak Software, Inc. Germany). All experiments were repeated 3 times.

### Calcium influx detection with Fluo 4-AM

The concentration of intracellular Ca^2+^ was determined using a cell-permeable fluorescent calcium indicator, Fluo 4-AM. Cells were treated with DMEM-F12 and 10% bovine serum for 24 h and washed 3 times with D-Hanks balanced salt solution without Ca^2+^ (Ca^2+^-free HBSS). Subsequently, cells were loaded with 2μmol/l Fluo 4-AM for 30 minutes at 37 °C in the dark, then washed 3 times with Ca^2+^-free HBSS to remove the extracellular Fluo 4-AM. The solution was replaced with HBSS with Ca^2+^ before testing. Imaging was performed using the Leica SP8 Confocal Inverted Microscopy (Leica, Mannheim, Germany) and analyzed with Image-Pro Plus 6.0. We used the measured average fluorescence intensity of each cell in the field (F), normalized to the non-specific background fluorescence (F0) to obtain the fluorescence intensity (F/F0)^22^.

### Western blot

Ishikawa and HEC-1A cells with different procedures were harvested and lysed, and Western blots were conducted as previously described^23^. Fifty micrograms of protein was subjected to SDS-PAGE and transferred into NC membranes. The membranes were incubated with corresponding antibodies, followed by horseradish peroxidase-conjugated secondary antibody. We use the ECL substrate to detect the protein expression. The band intensities were determined using the Bio-Rad imaging system (Hercules, CA, USA).

### Tandem Mass Tagging (TMT) proteomics analysis and bioinformatics analysis

Proteomics analysis of ishikawa cells was conducted by PTM-Bio labs Co. Ltd. (HangZhou, China). Protein sample processing includes protein preparation, trypsin digestion, TMT labeling, HPLC fractionation, LC–MS/MS analysis, and data analysis. The bioinformatics analysis of protein annotation, functional classification, functional enrichment, and cluster analysis were then performed.

### Immunofluorescence assay

For immunofluorescence, cells (2 × 10^5^ cells per well) were seeded in the coverslip in a 6-well plate with a coverslip inside. After a 24 h culture, cells were then fixed in 4% paraformaldehyde for 30 min and incubated in PBS with 0.1% Triton X-100 for 10 min on ice, and then blocked in 1% fetal bovine serum (FBS). Coverslips were moved to a piece of parafilm in a humid chamber, and 100 μL anti-paxillin (1:1000 dilution) was added on the coverslip. On the second day, cells were washed 3 times with PBS and we added 200 μl rhodamine phalloidin of 100 nM. They were then incubated at room temperature shielded from light for 30 min. Nuclei were stained with PBS with 2 μg/ml DAPI for 10 min. Staining was photographed by Leica SP8 Confocal Inverted Microscopy (Leica, Mannheim, Germany). All experiments were repeated 3 times

### Animal model of tumor xenograft

Four-week-old female BALB/C nude mice were purchased from the Charles River Company (Beijing Vital River Laboratory Animal Technology Co. Ltd) and were maintained in Specific Pathogen-Free (SPF) Laboratory Animal Center of Peking University People’s Hospital. All experiments were performed in accordance with the official recommendations of the Chinese animal community (2018PHC054). Endometrial cancer cell lines, ishikawa and HEC-1A xenografts were established in nude mice. Briefly, ishikawa cells with shNC, shTRPV4, and shTRPV4 + GSK1016790A, and HEC-1A cells with OE-NC, OE-TRPV4, and OE-TRPV4 + HC067047 were trypsinized and resuspended in PBS (pH 7.4) for injection into one mouse in a total volume of 200 μl. The suspension, containing 2 × 10^6^ cells, was injected into the abdominal cavity (5 mice for each group). On the 30th day after intraperitoneal injection, mice were sacrificed by cervical decapitation, and mice models died before being sacrificed were excluded. Peritoneal spreading and metastatic tumor numbers were then counted and photographed.

### Statistics analysis

Continuous variables were presented as mean ± SD. Categorized variables were shown as number (N) and percentages. Statistical differences between paired experimental groups were examined by two-tailed independent sample student’s *t*-test. or, in the case of more than two groups, one-way analysis of variance (ANOVA) with Bonferroni post hoc testing. Correlations between TRPV4 expression in different groups were analyzed by chi-square or Fisher’s exact tests. Mann–Whitney U test was used to analyze the difference between means. Differences were considered as significant for *p* < 0.05. Analyses were performed by SPSS 21.0 or Prism 7 software (GraphPad).

## Results

### Association between ionized calcium level and disease progression

**Fig. 1:**
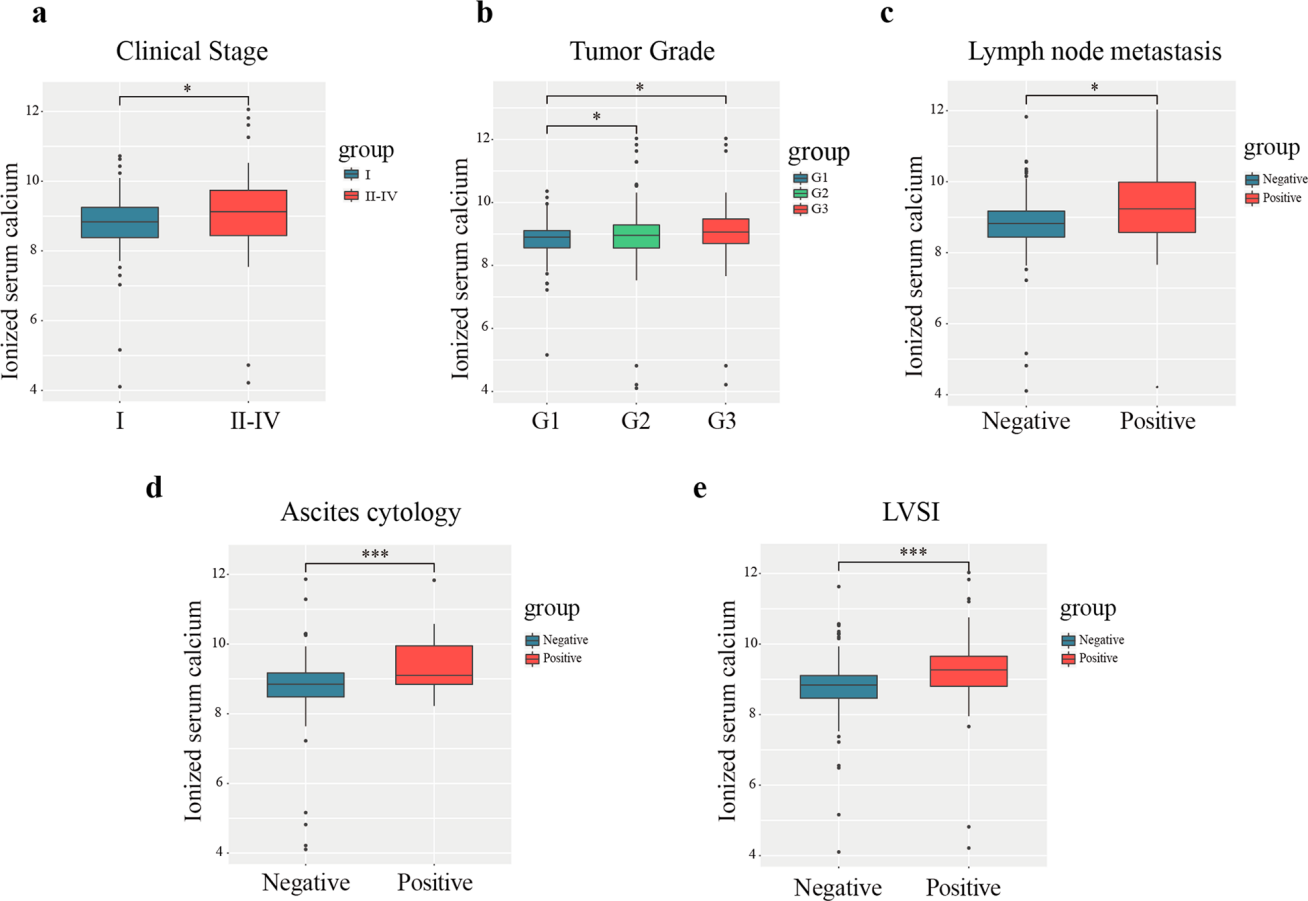
High level of serum ionized calcium was positively asscoiated with advanced endometrial cancer. Relationship between ionized serum calcium concentration and clinicopathological characteristics, including different **a** FIGO stage. **b** Grade. **c** Lymph node metastasis. **d** Ascites cytology. **e** LVSI. Wilcoxon signed-rank test and logistic regression were used to analyze the relationship between ionized calcium level and clinical pathologic features. *p < 0.05; **p < 0.01; ***p < 0.001. G, grade; LVSI, lymph-vascular space invasion.

A total of 510 patients were recruited to have their ionized calcium level and disease progression measured in this study. Participants’ mean age was 55.8 years (ranges 23–83 years) and the average BMI was 26.3 (range 17.2–60.8 kg/m^2^). Patients were histologically diagnosed as having either endometriod endometrial adenocarcinoma (EEA; *N* = 440) or other types (serous cancer, *N* = 48; clear cell cancer, *N* = 16, others, *N* = 6). The FIGO staging system was used to produce two groups: stage I (*N* = 406) and stage II–IV (*N* = 104). Detailed demographic information is shown in Table 1. As shown in Fig. 1, after adjusting for the serum albumin, serum ionized calcium level was significantly higher in the stage II–IV group compared to the stage I group (Fig. 1a). Moreover, ionized calcium was higher for patients with G 2/3 compared to G1 (Fig. 1b), patients who were positive for LNMs (Fig. 1c), patients with positive peritoneal cytology (Fig. 1d), and patients with LVSI positive status (Fig. 1e). Finally, in vitro experiments were conducted using transwell migration assays to verify the link between calcium concentration and cell migration (Fig. S1a, b): elevated migration was found in response to an extra increasing calcium concentration from 0 to 3 mM, and the calcium concentration of 4 mM and 5 mM did not increase the migration significantly (data not shown). Migration decreased in response to the calcium chelating agent BATPA-AM.

**Table 1:** Demographics of patients with endometrial carcinoma.

	Mean ± SD
Age	55.77 ± 9.56
BMI	26.32 ± 4.62
Serum calcium	9.18 ± 0.72
Ionized serum calcium	8.89 ± 0.69
Serum albumin	43.60 ± 4.19

	*N* (%)
Ascites cytology	
Negative	352 (90.96%)
Positive	35 (9.04%)
FIGO stage	
I	406 (79.61%)
II	25 (4.90%)
III	64 (12.55%)
IV	15 (2.94%)
Lymph node metastasis	
Negative	463 (90.78%)
Positive	47 (9.22%)
LVSI	
Negative	414 (83.30%)
Positive	83 (16.70%)
Tumor grade	
1	171 (33.53%)
2	220 (43.14%)
3	119 (23.33%)
Histology	
EEA	440 (86.28%)
Other types	70 (13.73%)

SD, standard deviation; BMI, body mass index; LVSI, lymph-vascular space invasion, EEA, endometriod endometrial adenocarcinoma; other types include serous endometrial cancer, mixed endometrioid and serous carcinoma, and clear cell carcinoma.

### TRPV4 was associated with EC and highly expressed in EEA

**Fig. 2:**
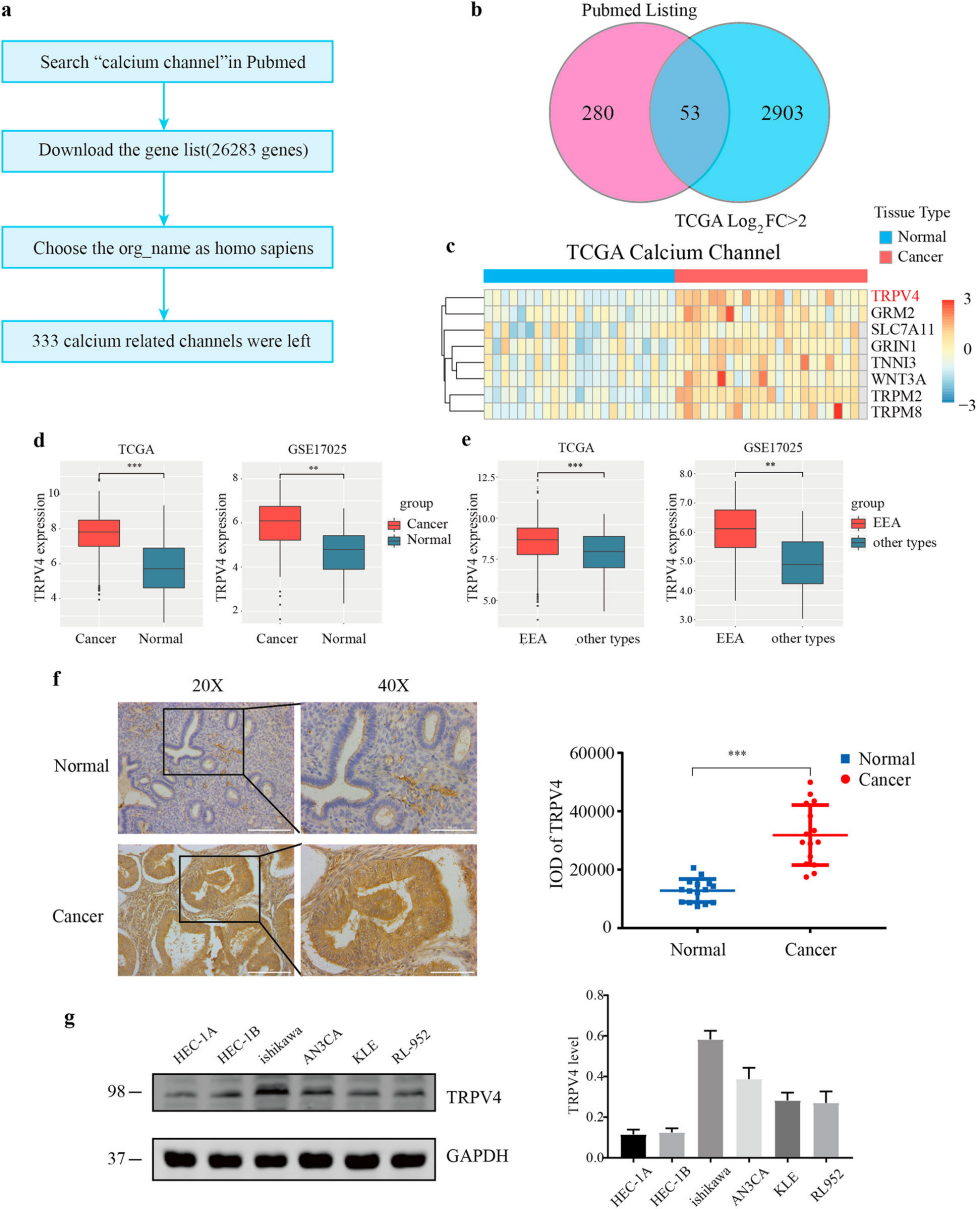
TRPV4 is associated with EC and highly expressed in endometriod endometrial adenocarcinoma. **a** Flow chart for calcium channel screening. **b** Venn diagram depicting the overlap between calcium channels in Pubmed (pink) and TCGA datasets (blue). **c** Heatmap of 8 up-regulated genes. **d** TRPV4 expression in cancer and normal tissues in TCGA and GEO datasets. **e** TRPV4 expression in different histological tissues in TCGA and GEO datasets. **f** Immunohistochemistry staining for TRPV4 in normal endometrial tissues and EC tissues, including images and a plot comparing TRPV4 IOD for normal and cancer conditions, Bars = means ± s.e.m., *n* = 15. **g** TRPV4 expression in 6 EC cell lines. EC, endometrial cancer. *, *p* < 0.05; **, *p* < 0.01; ***, *p* < 0.001.

Following confirmation that ionized calcium levels were related to EC progression, we next examined the link between EC and calcium channels. The selection flow chart was shown as Fig. 2a. In the Pubmed listing results, a total of 26283 genes containing “calcium channel”, and 333 genes belonged to homo sapiens. We intersected all the calcium channels in human (*N* = 333) with the calcium channels whose log_2_FC was greater than 2 in TCGA (*N* = 2956). Using this approach, 53 differentially expressed calcium channels were identified (Fig. 2b), including 8 up-regulated and 45 down-regulated genes in EC. Illustrative examples of 8 up-regulated genes were visualized in a heatmap in Fig. 2c. TRPV6 played an important role in EC during cervical invasion in our former study. Therefore, we concentrated on TRP family in the following study. Previous study had reported that TRPM2 played a pivotal role in EC, and preliminary experiments indicated that TRPM8 failed to promote metastasis in EC cell lines (Fig. S2a). We then analyzed EC and normal samples selected from TCGA and verified our findings using The NCBI GEO dataset GSE17025 that included TRPV4 expression data, and results were shown in Fig. 2d, e. These findings confirmed that TRPV4 was significantly higher in EC tissue compared to the normal group (*p* < 0.001), especially in the EEA group (*p* < 0.01).

Immunohistochemistry detection of TRPV4 was performed on 15 EC and 15 normal endometrial tissues. In Fig. 2f, the colors yellow and brown indicated a positive expression of the marker. TRPV4 was found to be expressed in the cytoplasm and membrane, and visual inspection suggested higher expression rates in the EC than normal group. In order to quantify this effect we assessed the integrated optical density (IOD) of the images, and found IOD was significantly higher in the EC group than that in the normal group.

For further detecting the function of TRPV4, endogenous TRPV4 protein levels were examined in 6 EC cell lines (HEC-1A, HEC-1B, ishikawa, AN3CA, KLE, and RL9-52) using Western blotting and immunofluorescence. Based on those results, we identified ishikawa cell line as having the highest level of TRPV4, and the HEC-1A cell line as having the lowest level of TRPV4 (Fig. 2g and Fig. S2b). AN3CA also had a relatively high expression of TRPV4, but the change in the expression of TRPV4 was not as significant as that in ishikawa cells after knockdown. The latter could better reflect the function of TRPV4. Therefore, ishikawa and HEC-1A cell lines were chosen for further analyses to create low and high TRPV4 expression conditions. Taken together, these results provided converging evidence that compared with other calcium channels, TRPV4 had a differentially important role in EC, and that compared to normal tissues, TRPV4 was elevated in EC in general, and in EEA in particular.

### Effect of TRPV4 depletion or overexpression on the influx of calcium and migration of EC cells in vitro and in vivo

The next set of analyses aimed to establish that TRPV4 was linked to both calcium regulation and cell migration in EC, in order to examine our hypothesis that TRPV4-related cancer progression was due to TRPV4-related calcium influx.

**Fig. 3:**
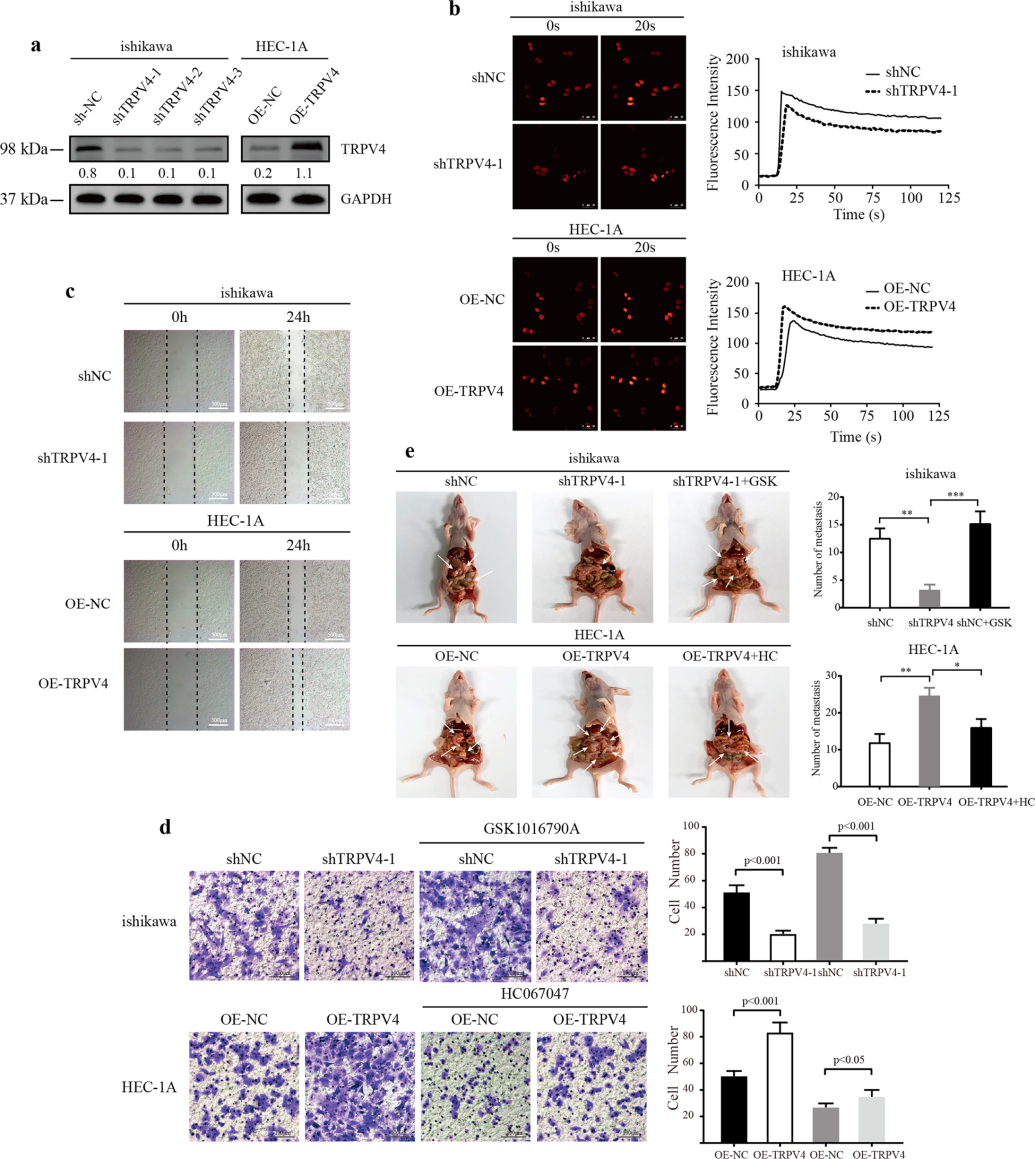
The effects of calcium concentration and TRPV4 expression (silencing or overexpressing) on EC cells. **a** The transfection efficiency of TRPV4 in ishikawa and HEC-1A cells, measured by Western blotting. **b** The effect of silencing or overexpressing TRPV4 on calcium influx. Depicted are representative confocal images after adding calcium channel activation KCl (60 mM). **c** Results of a wound-healing assay for ishikawa and HEC-1A cells after transfection for 24 h. **d** Results of transwell migration assay for ishikawa and HEC-1A cells after transfection for 24 h. Cells were stained with crystal violet (magnification, x200). **e** In vivo experiments used to verify the function of TRPV4 and its agonist (GSK1016790A), antagonist (HC067047). Numbers below the Western blot band, relative expression to GAPDH of each protein. *, *p* < 0.05; **, *p* < 0.01; ***, *p* < 0.001.

First, we established a mechanism for manipulating TRPV4 levels by using 3 shRNA (shTRPV4) vectors and one overexpression (OE-TRPV4) vector targeting TRPV4, which were transfected into Ishikawa (higher TRPV4 expression) and HEC-1A cells (lower TRPV4 expression), respectively. Ishikawa cells were also transfected with a negative control shRNA (shNC) vector, and HEC-1A cells with a negative control overexpression (OE-NC) vector. Western blotting and immunofluorescence were used to detect the transfection efficiency. All of the three shTRPV4 vectors significantly suppressed TRPV4 expression in the Ishikawa cell line and the expression of exogenous TRPV4 (OE-TRPV4) was efficiently up-regulated in HEC-1A cells (Fig. 3a and Fig. S3).

We next measured the levels of calcium influx as a result of suppression or overexpression of TRPV4, based on previous evidence that this influx occurred rapidly, within 3 minutes of TRPV4 expression changes. We used the cytoplasmic Ca^2+^ specific indicator, Fluo 4-AM, which was applied using confocal microscopy to assess the changes in calcium. For suppression and overexpression conditions, the peak value of calcium influx was about 20 s after the application of a KCl solution to activate the calcium channel. Compared with the control group, the peak of dose-dependent Fluo-4 fluorescence was lower in the shTRPV4 group and higher in the OE-TRPV4 group, confirming that TRPV4 content was associated with calcium influx (Fig. 3b).

Next, we examined whether TRPV4 affected the migration of cells in the ishikawa and HEC-1A cell lines. We used a gap closure assay to assess evidence of migration over a 24 h period, in different cell lines and under conditions of TRPV4 suppression or overexpression. The gap closure suggested that in the ishikawa cell line, cells in the suppression group (shTRPV4) had less motility than the corresponding control cells (shNC). Likewise, the motility of HEC-1A cells was enhanced by TRPV4 overexpression (OE-TRPV4) compared to the control condition (OE-NC) (Fig. 3c). In order to further investigate the effect of modifying TRPV4 levels on cell migration, transwell migration assay was employed in which a TRPV4 agonist (GSK1016790A, GSK) was added to ishikawa cells (shNC or shTRPV4 conditions) and its antagonist (HC067047, HC) was added to HEC-1A cells (OE-NC or OE-TRPV4 conditions). Consistent with the gap closure results, after 24 h, motility was significantly decreased in the shTRPV4 group compared to the shNC group. The addition of GSK increased motility in the shNC group, but not the shTRPV4 group. Similarly, in the HEC-1A line, cell motility was higher in the OE-TRPV4 than OE-NC group. Adding the antagonist HC led to decreased motility in the OE-NC group compared to the OE-TRPV4 group (*p* < 0.05; Fig. 3d). Taken together, these findings clearly demonstrated that increased TRPV4 expression is associated with more cell migration in both ishikawa and HEC-1A cells.

Following the in vitro findings described above, we examined the in vivo effect of TRPV4 by injecting shNC, shTRPV4, shTRPV4+GSK, and OE-NC, OE-TRPV4, OE-TRPV4 + HC cells into abdomens of nude mice (*N* = 5 per condition). As expected, there were significantly less metastatic peritoneal nodules in the shTRPV4 group compared with the shNC group, but the number increased in shTRPV4+GSK group. Likewise, more peritoneal spreading was observed in the OE-TRPV4 group compared with OE-NC group, and metastatic sites declined in OE-TRPV4 + HC group (Fig. 3e). These in vivo data were consistent with the in vitro results and confirmed that TRPV4 promoted EC cell migration.

### Identification of functions regulated by TRPV4 via proteomic analysis

**Fig. 4:**
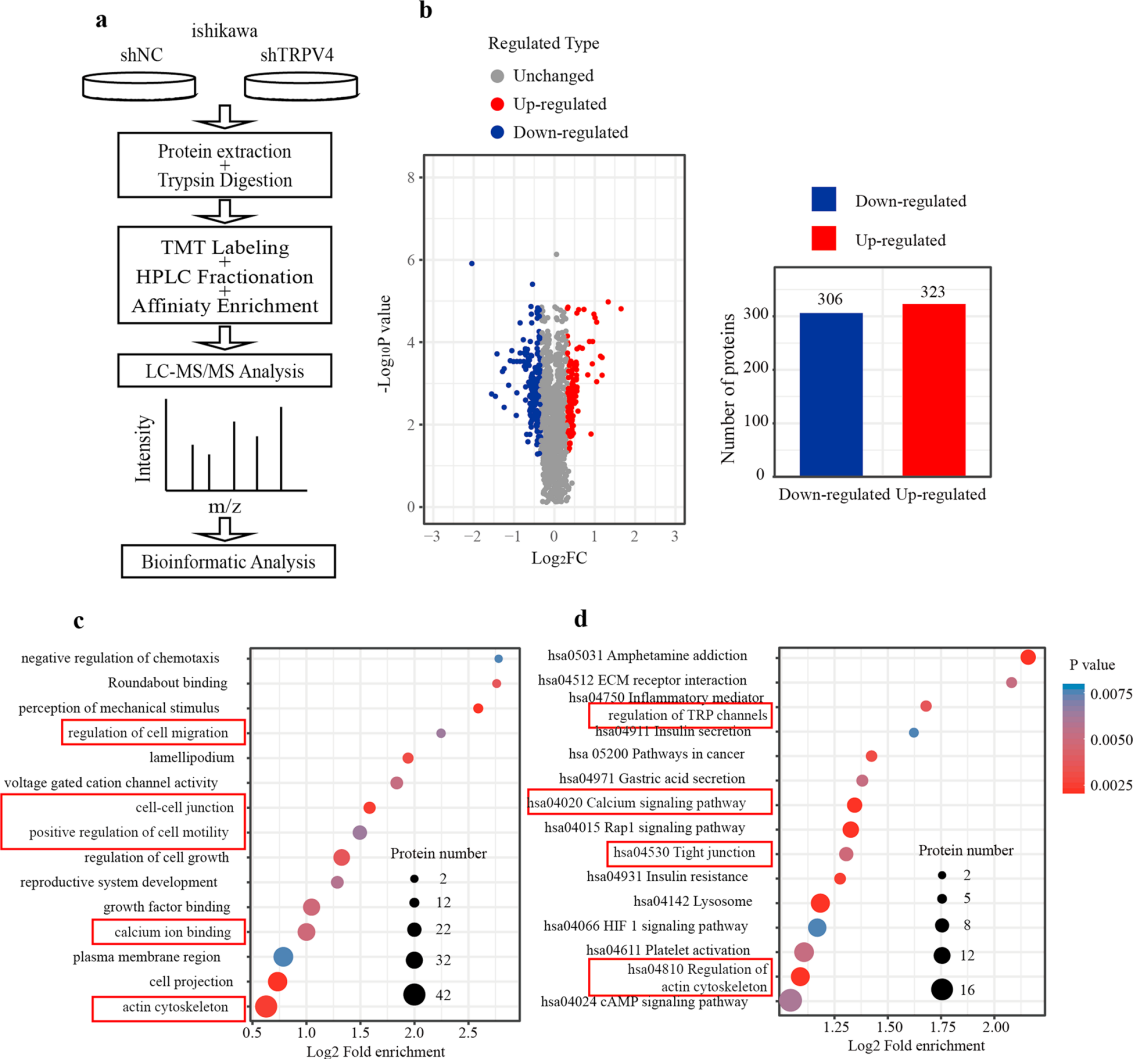
Proteomic analysis of shNC and shTRPV4 groups in the ishikawa cell line. **a** Flow diagram of proteomic analysis. **b** Volcano chart and column chart show the distribution of genes up- and down-regulated in the shTRPV4 group. **c**, **d** Results of GO and KEGG analysis of the up-regulated genes.

Given previous findings from other types of cancer, we next verified that TRPV4 acted to instigate cell migration in EC via its effects on the actin cytoskeleton. We tested for this and other TRPV4 regulatory mechanisms in ishikawa cells using proteomic analysis to measure the expression of a range of proteins in the shNC and shTRPV4 groups. The flow diagram of the method was presented in Fig. 4a, and this analysis identified 6069 proteins, of which 5265 were quantified.

In order to test for TRVP4-related differential gene expression, we used a threshold of log_2_FC greater than 1.5 (*p* < 0.05), which identified 629 proteins, including 323 up-regulated in the shTRPV4 compared to shNC group, and 306 down-regulated (Fig. 4b). Gene Ontology (GO) analysis was conducted to classify the differentially expressed proteins into 3 categories: cell component (CC), molecular function (MF), and biological process (BP). Results suggested that differentially expressed genes were involved in the regulation of multiple biological processes including “regulation of cell migration”, “cell–cell junction”, “positive regulation of cell motility”, “calcium ion binding”, and “actin skeleton” (Fig. 4c). Figure 4d showed the KEGG pathway analysis for the differentially expressed proteins. Results revealed that these proteins were involved in pathways associated with “regulation of TRP channels”, “calcium signaling pathway”, “tight junction”, and “regulation of the actin cytoskeleton”. The GO and KEGG analysis results were also verified using the TCGA dataset, which revealed a similar pattern (Fig. S4a, b). These results provided evidence that TRPV4 promoted cell migration via modulation of the actin cytoskeleton.

### TRPV4 increased EC progression by mediating filament organization and anchored component through Rho protein pathway

**Fig. 5:**
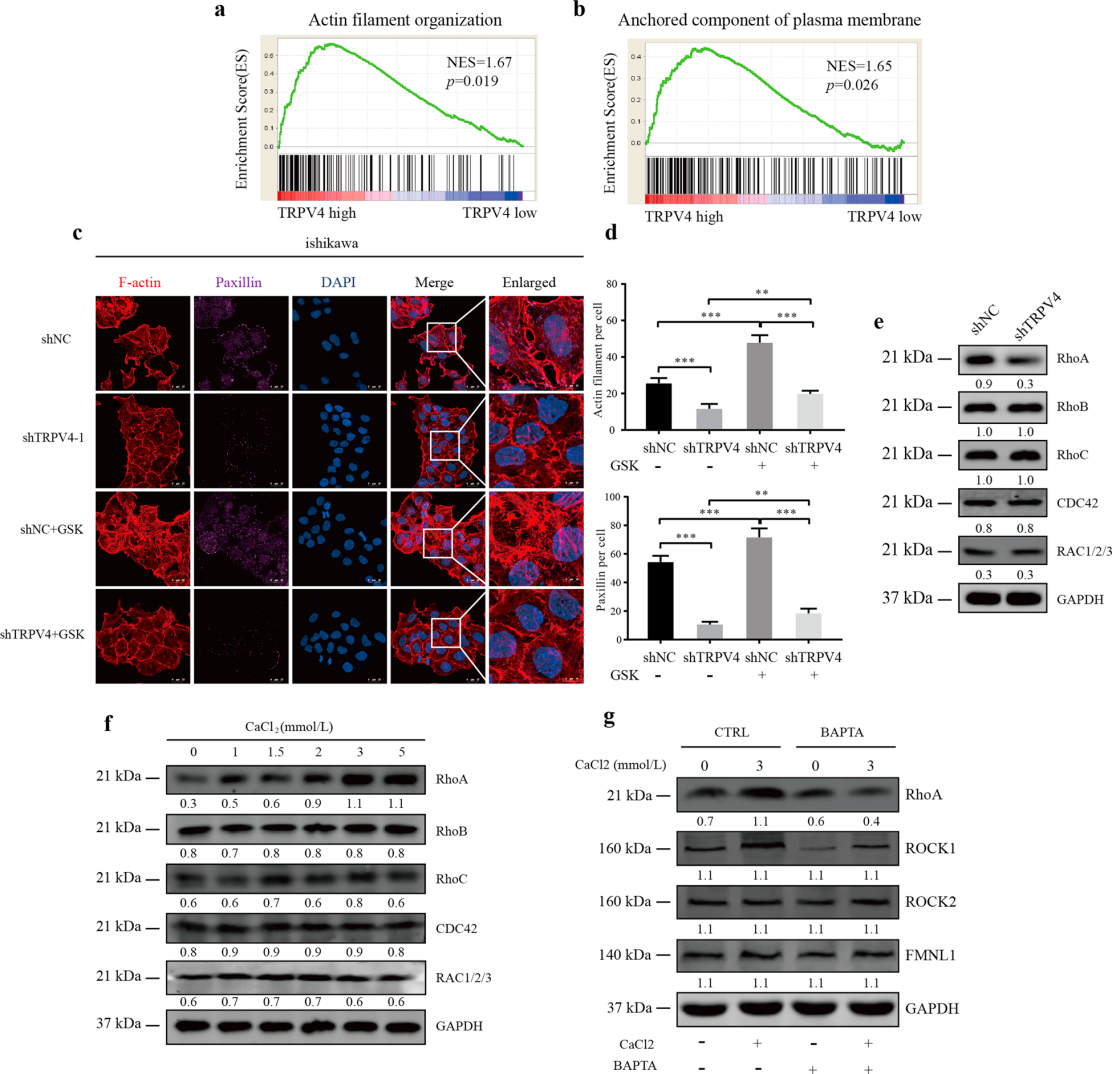
TRPV4 activated cytoskeleton and Rho protein pathway in EC. **a**, **b** GSEA analysis demonstrated high expression of TRPV4 is enriched in actin filament organization and anchored component of plasma membrane. **c** The results of cytoskeleton assay of ishikawa cells visualized by confocal microscopy, with representative images shown. Cell nuclei were stained with DAPI. Magnification 63×. Scale bar: 25 or 10 μm. **d** Statistical numbers of actin filaments and PXN in each group. **e** TRPV4 silencing and the expression of Rho family GTPase pathway in ishikawa cell. **f** Relationship between calcium concentration and Rho family GTPase pathway. **g** Calcium chelating agent BAPTA-AM (10 μM) significantly inhibited the RhoA-ROCK1 pathway. Columns represent mean ± SEM. Numbers below the Western blot band, relative expression to GAPDH of each protein.

In our next set of analyses, we conducted Gene Set Enrichment Analysis (GSEA) to characterize the differences between EC samples from the TCGA dataset which we identified as low or high in TRPV4 expression based on median expression values. Results indicated that in the high compared to low TRPV4 expression samples, the genes were enriched in actin filament organization (NES = 1.67, *p* = 0.019) and the anchored component of the plasma membrane (NES = 1.65, *p* = 0.026), a category that included the adaptor protein paxillin (PXN), which played an important role in cell migration (Fig. 5a, b).

Following these results, we used immunofluorescent staining of EC cells to evaluate the levels of two cytoskeletal proteins, actin filament (F-actin) and PXN, in the TRPV4-depletion (shTRPV4) and overexpression (OE-TRPV4) groups and their control groups. In the shNC group of ishikawa cells, the F-actin was well modeled and distributed, with actin filament accumulating in the leading edge. In contrast, in the shTRPV4 group, there were less actin filament and PXN, and it was disordered in the arrangement. After adding the TRPV4 agonist GSK, in both the shNC and shTRPV4 groups the actin fiber and PXN levels increased significantly, and the actin fiber structure became thicker and more ordered. But the actin fiber in shTRPV4+GSK did not become thickened and ordered because TRPV4 in this group was knocked down, and GSK could not specifically stimulate TRPV4 and its downstream activity (Fig. 5c, d). In the HEC-1A line, both F-actin and PXN levels were lower for the OE-NC compared to the OE-TRPV4 group. Adding the TRPV4 antagonist HC reduced both actin and PXN levels in OE-NC and OE-TRPV4 groups (Fig. S5a–c). These results in combination with the results from the proteomic, GO, and KEGG analyses, provide strong evidence that TRPV4 affects cytoskeletal rearrangement, including F-actin and PXN levels.

### TRPV4 and calcium influx promotes activation of the RhoA/ROCK1 pathway

Because one of the most common mechanisms for TRP channels to mediate actin was via Rho GTPases, we next examined the link between TRPV4 and Rho expression. First, according to the GSEA results (Fig. S5d) comparing low and high TRPV4 expression samples in EC, high TRPV4 expression was enriched in Rho protein signal transduction (NES = 1.52, *p* = 0.032). Next, we used Western blotting with the ishikawa cell line and found that the expression of RhoA decreased in the shTRPV4 group (Fig. 5e), but this relationship to TRPV4 was not found in other Rho pathway proteins.

Our findings thus far suggested that TRPV4 had its effect on cytoskeletal structure and metastasis in EC via regulating extracellular Ca^2+^. To further examine this conclusion, we assessed the relationship between the concentration of calcium and the Rho protein signal pathway. We extracted whole protein from the ishikawa cells, and the results of Western blotting suggested that the expression of RhoA was initiated by a high concentration of CaCl_2_ and increased as the concentration of CaCl_2_ increased (Fig. 5f, g). We then examined the downstream components of the RhoA pathway, including ROCK1, ROCK2, and Formins1 (FMNL1). These analyses demonstrated that CaCl_2_ played a role in the ROCK1 pathway, with increased expression of ROCK accompanying higher calcium concentration; additionally, the use of the calcium chelating agent BAPTA-AM blocked the function of CaCl_2_. These findings confirm that TRPV4 and calcium influx activated the RhoA/ROCK1 pathway in EC cells, triggering the mechanisms underpinning cell migration.

### LIMK/cofilin signaling pathway was involved in calcium- and TRPV4-mediated activation, regulating migration and cytoskeleton in EC cells

**Fig. 6:**
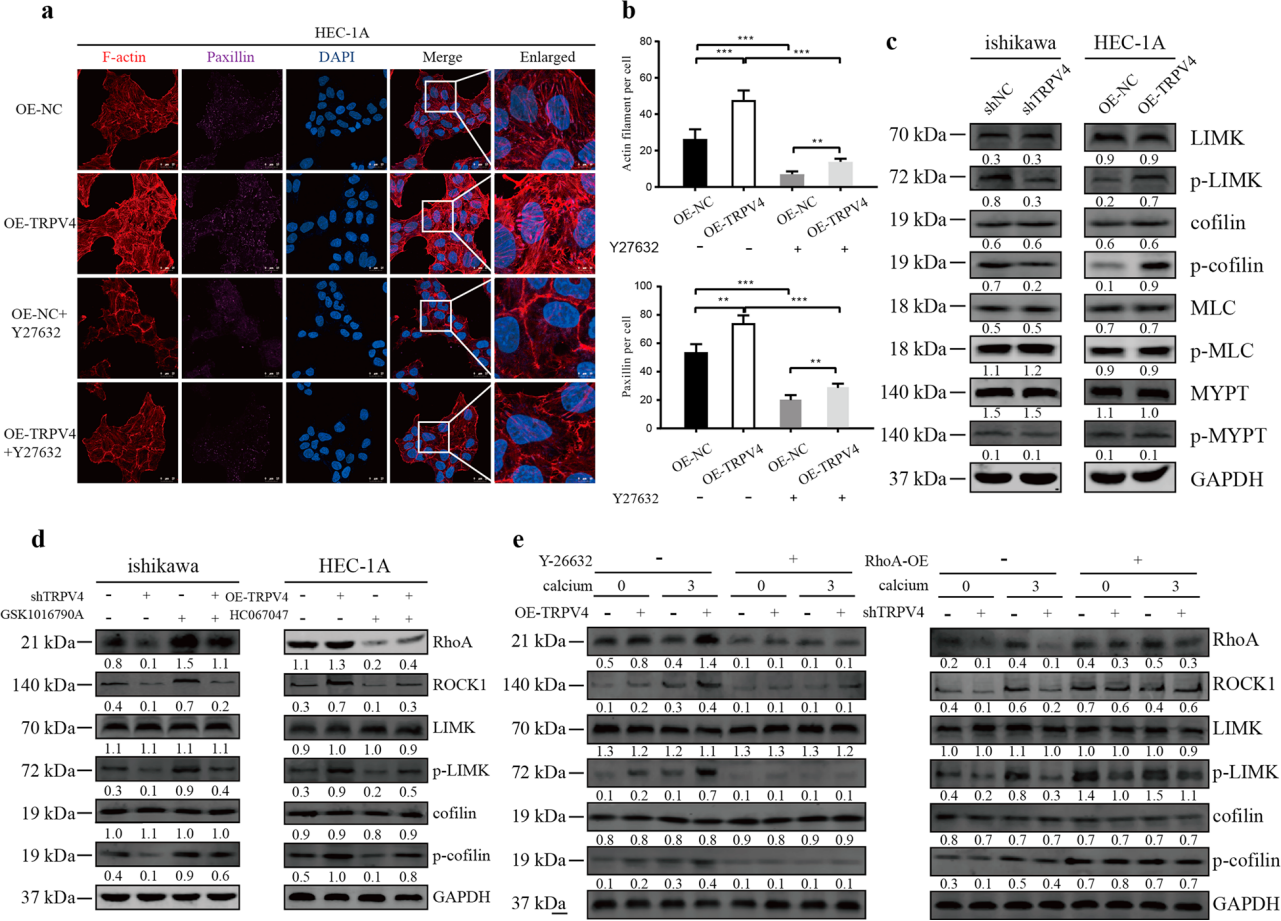
RhoA-ROCK1 mediated cytoskeleton through LIMK-cofilin pathway. **a** Influence of Y27632 (10 μM) on the F-actin and PXN. Magnification 63×. Scale bar: 25 or 10 μm. **b** Statistical number of F-actin and PXN. **c** Association between TRPV4 expression and the downstream of ROCK1, including LIMK, cofilin, MLC, MYPT. **d** TRPV4 affected the expression of the RhoA-ROCK1- LIMK-cofilin pathway and these effects were amplified by TRPV4 agonist GSK1016790A (50 nM) or decreased by TRPV4 antagonist HC067047 (10 μM). **e** The synthetic effect of calcium, TRPV4, and RhoA on the RhoA-ROCK1- LIMK-cofilin pathway, Y-26632, RhoA antagonist. Columns represent mean ± SEM. Numbers below the Western blot band, relative expression to GAPDH of each protein.

In the next set of analyses, we explored the function of the RhoA protein in mediating actin cytoskeleton and PXN. In the HEC-1A cells, F-actin and PXN levels were higher for the OE-TRPV4 compared to OE-NC group, and levels of actin and PXN were reduced following the addition of the RhoA antagonist Y27632 in NC-OE group, This effect could be reversed by overexpressing RhoA (Fig. 6a, b). Likewise, the results of a cytoskeletal assay of ishikawa cells revealed that the level of actin filament and PXN were lower in shTRPV4 compared with the shNC group, as previously found. But the overexpression of RhoA (OE-RhoA) increased the level of both actin and PXN in both shNC and shTRPV4 groups (Fig. S6a–c). The role of RhoA was further confirmed by examining its effect on migration: in the ishikawa cells, the shTRPV4 group demonstrated less migration compared to shNC group (Fig. S6d), but migration was increased in the OE-RhoA condition. Finally, in the HEC-1A cells, more migration was observed in the OE-TRPV4 compared to the OE-NC group, and migration was reduced in both groups following RhoA inhibition (Y27632 condition; Fig. S6e). These results confirmed that the level of RhoA expression was strongly linked to the expression of cytoskeletal proteins and cell migration, which can be influenced by directly manipulating RhoA levels. What’s more, RhoA was also a downstream molecule for TRPV4, and the mechanism of TRPV4 regulating cytoskeleton is through RhoA pathway.

RhoA was known to activate ROCK1, therefore we then examined the role of downstream components of ROCK1, including LIMK, cofilin, MLC, MYPT, and its respective phosphorylated molecules in both differentially expressed TRPV4 cells and altered calcium concentration cells. The results suggested that the expression of p-LIMK and p-cofilin altered in response to different expression and activity of TRPV4, confirming their influence on the LIMK/cofilin pathway (Fig. 6c, d).

**Fig. 7:**
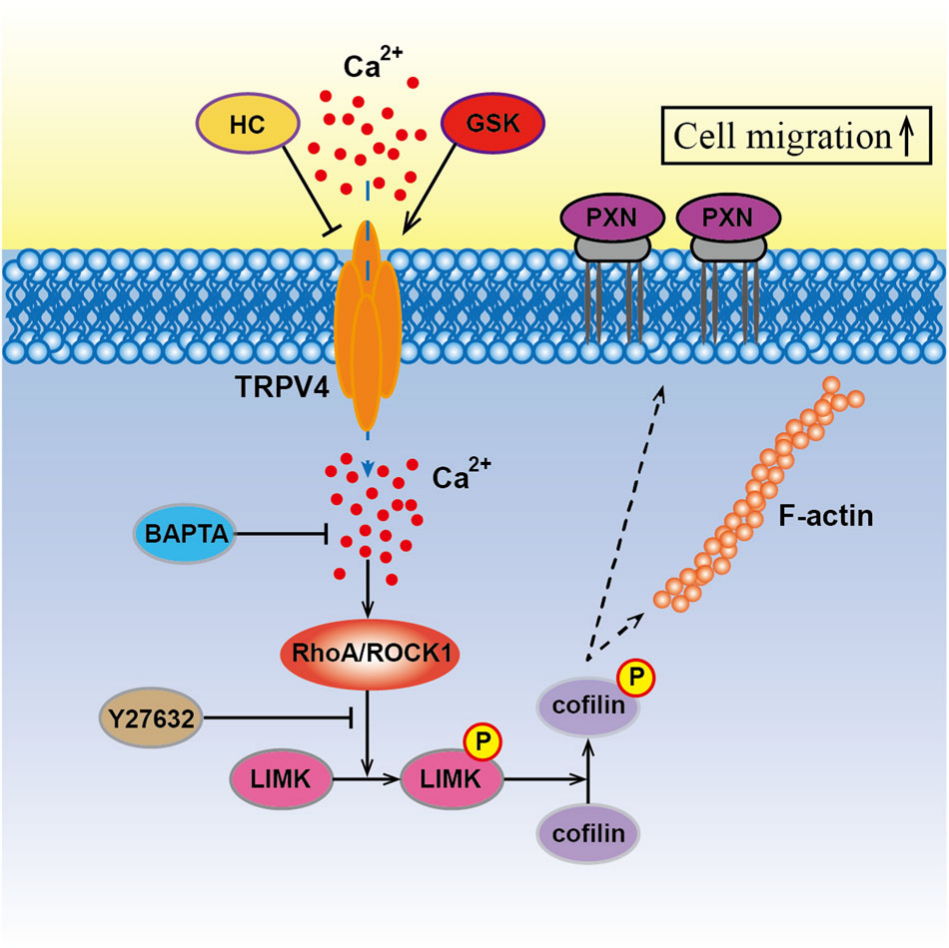
Signaling cascade for the aberrant TRPV4 and calcium activation in endometrial cancer.

To further identify how TRPV4 and calcium influx affected the LIMK/cofilin pathway, we examined the expression of the Rho-ROCK1- LIMK-cofilin pathway in both ishikawa and HEC-1A cell lines. As shown in Fig. 6e, the level of RhoA, ROCK1, p- LIMK, and p-cofilin increased with the rising expression of TRPV4 and calcium concentration. After overexpression of RhoA, the downstream protein level of the whole pathway increased, but the effect of calcium and TRPV4 level on the downstream components disappeared. Similar phenomena were observed in the RhoA with Y27632 condition. Co-localization of F-actin and cofilin was shown in Fig. S6f, g. Above all, these results demonstrated that TRPV4 regulated the cytoskeletal structure and mechanisms of metastasis through LIMK/cofilin pathway (Fig. 7).

## Discussion

Distant metastasis was a critical factor determining EC prognosis^3^, and there were mounting evidences that calcium influx played an essential role during metastasis^10^. However, the molecular mechanisms of metastasis of EC still remained unclear, in terms of both the specific calcium channels involved, and the downstream mechanisms that were triggered by calcium influx, which lead to cell migration. The current study provided insight on both these issues. We demonstrated for the first time that the expression of the calcium channel TRPV4 was related to ionized calcium levels, prognosis, and cell metastasis in EC. Additionally, we provided evidence for the causal chain of mechanisms leading from increased TRPV4 expression and calcium influx to the modulation of the RhoA/ROCK1/LIMK/cofilin pathway, and ultimately to changes in cytoskeletal actin that induced cell migration.

Calcium and its related channels had been reported to promote tumor progression depending on different tumors^24^. In the serum, it was the ionized calcium that played a physiological role but not the total calcium, and the rise of cytoplasmic ionized calcium concentration was an important factor in cellular signal transduction^25^. Previous studies showed that calcium signaling elicited many biological responses, and aberrant calcium signaling was involved in different pathogenesis^26^. The mechanism underlying calcium-activated movement remained unknown in EC, which was in part controlled by confined calcium pulses that promoted local lamellipodia retraction and adhesion cycles along the leading edge of moving cells^27^. As a non-selective calcium ion channel, TRPV4 was widely expressed in endothelial cells, which acted as a mechanosensor of cyclic strain and shear stress in endothelial cells^28,29^. TRPV4 promoted cancer progression through multiple regulations. Previous research had found that TRPV4 expression was dysregulated in some types of cancers^30^, for example, TRPV4 had been found to be overexpressed in metastatic breast cancer^31^, and a recent study implicated that TRPV4 also enhanced cell migration to the lung^32^. TRPV4 regulates matrix stiffness and TGFβ1-induced epithelial-mesenchymal transition (EMT)^33^, and it was also involved in malignant biological behavior of hepatocellular carcinoma via modulation of ERK signaling pathway^34^. TRPV4-mediated calcium entry might also promote gastric cancer progression in a calcium signaling-dependent manner^35^. It not only played a critical role in cancer progression, but also in bone marrow adipocyte remodeling induced by leukemia cells and can be activated by bladder stretch through coupling to a rigid intracellular and intercellular structural network^36,37^. But its role in EC still remained elusive. Therefore, we explored the function and mechanism of calcium and TRPV4 in EC. Our findings revealed that high ionized calcium concentration was related to a more advanced clinical stage or grade of EC, and TRPV4 was found to be the key channel in EC as well. Although the biofunction of TRPV4 in cancers was only preliminarily explored, no previous research had examined the role of TRPV4 in cell migration or invasion in EC. We used bioinformatics and immunohistochemistry to demonstrate that TRPV4 was abnormally regulated in EC. Furthermore, we chose the high- or low-TRPV4-expressed EC cell lines, ishikawa and HEC-1A to vertify the biological function of TRPV4 in endometrioid endometrial adenocarcinoma (EEA).”

In the current study, manipulations of TRPV4 depletion and overexpression were used to demonstrate the significant correlation between TRPV4 expression and EC cell migratory capacity. TRPV4 depletion decreased calcium influx in ishikawa cells and overexpression increased calcium levels in HEC-1A cells. The metastatic ability was diminished in ishikawa cells accompanied by TRPV4 depletion. When TRPV4 was overexpressed in HEC-1A cells, the opposite data were obtained. Calcium ions increased the expression of TRPV4 by VPAC1, CaSR, and Ca^2+^-activated K^+^ channels including SK1/3, IK1, and BK in other cancers, but the mechanism of overexpressed TRPV4 in EC still remained elusive^11,35,38^. As a potent intracellular second messenger, the elevation of serum calcium might be associated with metabolic syndrome in EC^39^, and increased calcium influx promoted the stiffness of tissue^40^. Moreover, TRPV4 was a mechanosensitive channel, and the expression of TRPV4 was regulated by tissue stiffness^32,41^. TRPV4 availability with agonists or antagonists was conducted as well, and we found that the TRPV4 agonist GSK enhanced TRPV4 function and rescued the motility inhibited by knockdown. In recent years, the roles of TRPV4 in cell proliferation, differentiation, and migration had been extensively studied. Its abnormal expression had also been closely related to the onset and progression of multiple tumors. Therefore, TRPV4 might be a target for cancer diagnosis and treatment^42^. We found that TRPV4-induced migration was attenuated by blocking calcium. The in vivo study demonstrated that the numbers of metastatic peritoneal nodules were significantly influenced by TRPV4. These findings, which linked calcium levels with EC progression, was supported by previous studies that calcium influx caused by the overexpression of TRPV4 were related to carcinogenesis^43^, metastasis^44^, and that serum calcium increased the risk of breast cancer^45^ and gastrointestinal cancer^46^.

Given that migratory capability in TRPV4 had been found in vitro and in vivo, the study of the role and mechanism of TRPV4 in the migration in EC cells had important clinical values. To further investigate the downstream pathway of TRPV4, we conducted the proteomics and bioinformatics analysis. Proteomic and GSEA analysis results indicated that differentially expressed genes in EC were distributed in the actin cytoskeleton and involved in cytoskeletal function, including actin filament organization and anchored component of the plasma membrane.

The various components of the cytoskeleton, actin (microfilaments), microtubules (MTs), and intermediate filaments, were highly integrated and their functions were well orchestrated in normal cells. In contrast, mutations and abnormal expression of cytoskeletal-associated proteins played an important role in the ability of cancer cells, such as resisting chemotherapy, proliferation, and migration^47,48^. Increased migratory ability was generally associated with high cytoskeletal tension, accompanied by lamellipodia formation and an elevated number of anchored components^49^. In the current study, the results revealed that actin filament and paxillin (PXN) were positively regulated by TRPV4 and its agonist GSK. The structure and number of cytoskeleton were significantly diminished by TRPV4 antagonist HC067047. Previous studies also found that TRPV4 regulated the thickness of F-actin stress fibers and the reinforcement of focal adhesion contacted through TRPV4-induced cation/calcium influx^50^. Many signaling pathways have been reported to have an impact on cytoskeletal regulation through metastasis including RhoA/ROCK, PI3K/mTOR, and Hippo pathway^51^. In this research, we focused on the RhoA/ROCK1 pathway and cytoskeletal regulation. Studies on the role of actin and its interacting partners have highlighted key signaling pathways, RhoA/ROCK and its downstream effector proteins that, through the cytoskeleton, mediated tumor cell migration, invasion and metastasis. It was reported that RhoA regulated its major target ROCK1^52^, and the net effect of ROCK1 activation was to enhance actin cytoskeleton and increase actin-myosin–dependent force generation. Increased ROCK1 was correlated with poor clinical outcomes and late-stage tumors^53^. The LIM domains enabled the LIM kinases to interact directly with many macromolecular partners, including ROCK1 and ROCK2. As the direct downstream of LIMK, cofilin-mediated severing accelerated the turnover and spatial reorganization of F-actin^54^. Our study found that the activity of RhoA was increased with TRPV4 expression, and the calcium concentration. The RhoA/ROCK1 signaling pathway was blocked by calcium chelating agent BAPTA-AM. Based on these results, we could conclude that TRPV4 regulated the thickening of F-actin stress fibers and the reinforcement of focal adhesion contacted through TRPV4-induced cation/calcium influx through the RhoA/ROCK1 signaling pathway.

In the downstream of ROCK1, LIMK and cofilin were crucial regulators. Actin reorganization was reregulated by phosphorylated-cofilin (p-cofilin). Protein phosphorylation has been demonstrated to be critical for tumor cell proliferation, cell survival and signal transduction^55^. As an important post-translational modification, phosphorylation could regulate protein activity^56^. The phosphorylation site of cofilin localized in the actin-binding domain and inhibits its binding to actin filaments, completely blocking cofilin ability to the promotion of filament disassembling. Therefore, the activity of cofilin regulated by its phosphorylation status determined the cytoskeleton dynamics. Previous research revealed that LIMK promoted the migration of cancer through regulating the phosphorylation of cofilin^57^. Moreover, we examined the role of RhoA on the migration and cytoskeleton. The results showed that overexpression of RhoA stimulated cell migration in ishikawa cells, and inhibition of RhoA blocked migration in HEC-1A cells by the regulation of cytoskeletal proteins, F-actin, and PXN. The signaling pathway of cytoskeletal regulation by TRPV4 and calcium influx might be through RhoA/ROCK1/LIMK/cofilin. These findings were in keeping with previous evidence that LIMK and cofilin were crucial regulators of actin as part of the RhoA/ROCK/LIMK/cofilin pathway. RhoA activation increased ROCK1 expression, which in turn activated LIMK. This led to direct phosphorylation of cofilin^58^, inhibiting its actin-depolymerizing activity and enabling stabilization of the actin stress fibers^58^. Taken together these findings provided evidence that the well-established RhoA/ROCK1/LIMK/cofilin pathway was involved in TRPV4- and calcium-induced cell migration.

In summary, we had demonstrated a novel link in EC between TRPV4 and cell migration. This link was found by clinical features of EC patients and explained by a series of causal mechanisms driven by TRPV4-induced calcium influx and traced through the RhoA/ROCK1/LIMK/cofilin pathway to induce motility-related changes in the actin cytoskeleton. These findings should motivate future research on the role of TRPV4 in cell migration in EC, as our results indicated that TRPV4 induced its metastatic capacities, and was, therefore, a strong candidate for developing targeted therapies.

## Conclusion

Our findings indicated that serum ionized calcium is significantly associated with poor prognosis in EC. TRPV4 and calcium-induced metastasis by regulating cytoskeleton through RhoA/ROCK/LIMK/cofilin pathway and TRPV4 should be targeted to improve therapeutic strategies in EC. TRPV4 also had the potential to be targeted pharmacologically for EC therapy and prevention.

## Supplementary information

Supplementary Figure S1-S4

Supplementary Figure S5-S6

Supplementary Figure Legends

## References

[1] Siegel RL, Miller KD, Jemal A (2017). Cancer Statistics, 2017. CA Cancer J. Clin..

[2] Chen W (2016). Cancer statistics in China, 2015. CA Cancer J. Clin..

[3] Morice P, Leary A, Creutzberg C, Abu-Rustum N, Darai E (2016). Endometrial cancer. Lancet.

[4] Li X (2019). An elevated preoperative serum calcium level is a significant predictor for positive peritoneal cytology in endometrial carcinoma. Chin. J. Cancer Res..

[5] Li XC (2020). Increased serum calcium level promotes the risk of lymph node metastasis in endometrial cancer. Cancer Manag. Res..

[6] Stewart TA, Yapa KT, Monteith GR (2015). Altered calcium signaling in cancer cells. Biochim. Biophys. Acta.

[7] Jardin I, Rosado JA (2016). STIM and calcium channel complexes in cancer. Biochim. Biophys. Acta.

[8] Middelbeek J (2012). TRPM7 is required for breast tumor cell metastasis. Cancer Res..

[9] Hasna J (2018). Orai3 calcium channel and resistance to chemotherapy in breast cancer cells: the p53 connection. Cell Death Differ..

[10] Li N (2018). The role of BKCa in endometrial cancer HEC-1-B cell proliferation and migration. Gene.

[11] Xie R (2017). Calcium promotes human gastric cancer via a novel coupling of calcium-sensing receptor and TRPV4 channel. Cancer Res..

[12] Hao J (2015). Ca2+ channel subunit alpha 1D promotes proliferation and migration of endometrial cancer cells mediated by 17beta-estradiol via the G protein-coupled estrogen receptor. FASEB J..

[13] Fiorio Pla A, Gkika D (2013). Emerging role of TRP channels in cell migration: from tumor vascularization to metastasis. Front Physiol..

[14] Canales J (2019). A TR(i)P to Cell Migration: New Roles of TRP Channels in Mechanotransduction and Cancer. Front Physiol..

[15] Becker D, Bereiter-Hahn J, Jendrach M (2009). Functional interaction of the cation channel transient receptor potential vanilloid 4 (TRPV4) and actin in volume regulation. Eur. J. Cell Biol..

[16] Xiang T (2016). DACT2 silencing by promoter CpG methylation disrupts its regulation of epithelial-to-mesenchymal transition and cytoskeleton reorganization in breast cancer cells. Oncotarget.

[17] Fife CM, McCarroll JA, Kavallaris M (2014). Movers and shakers: cell cytoskeleton in cancer metastasis. Br. J. Pharm..

[18] Lee WH (2016). TRPV4 Regulates Breast Cancer Cell Extravasation, Stiffness and Actin Cortex. Sci. Rep..

[19] Zhao ZS, Manser E (2005). PAK and other Rho-associated kinases-effectors with surprisingly diverse mechanisms of regulation. Biochem. J..

[20] Sun L, Wang J, Zhang L, Li X, Shen D (2013). Expression of ER-alpha36, a novel variant of estrogen receptor in endometrial carcinoma and its clinical significance. Gynecol. Obstet. Invest..

[21] Bao XX (2012). Nifedipine induced autophagy through Beclin1 and mTOR pathway in endometrial carcinoma cells. Chin. Med J. (Engl.).

[22] Sun R, Yang Y, Ran X, Yang T (2016). Calcium influx of mast cells is inhibited by aptamers targeting the first extracellular domain of orai1. PLoS ONE.

[23] Zhang L (2009). Nongenomic effect of estrogen on the MAPK signaling pathway and calcium influx in endometrial carcinoma cells. J. Cell Biochem..

[24] Bortolato B (2017). Depression in cancer: the many biobehavioral pathways driving tumor progression. Cancer Treat. Rev..

[25] Tsuji Y (2002). Cancer cell contact causes oscillatory and polarized rise of cytoplasmic ionized calcium concentration in human umbilical vein endothelial cells. Int. J. Oncol..

[26] Fiorio Pla A (2012). TRPV4 mediates tumor-derived endothelial cell migration via arachidonic acid-activated actin remodeling. Oncogene.

[27] Tsai FC, Meyer T (2012). Ca2+ pulses control local cycles of lamellipodia retraction and adhesion along the front of migrating cells. Curr. Biol..

[28] Baylie RL, Brayden JE (2011). TRPV channels and vascular function. Acta Physiol. (Oxf.).

[29] Mendoza SA (2010). TRPV4-mediated endothelial Ca2+ influx and vasodilation in response to shear stress. Am. J. Physiol. Heart Circ. Physiol..

[30] Hope JM, Greenlee JD, King MR (2018). Mechanosensitive ion channels: TRPV4 and P2X7 in disseminating cancer cells. Cancer J..

[31] Lee WH (2017). TRPV4 plays a role in breast cancer cell migration via Ca(2+)-dependent activation of AKT and downregulation of E-cadherin cell cortex protein. Oncogenesis.

[32] Cappelli HC (2019). Mechanosensitive TRPV4 channels stabilize VE-cadherin junctions to regulate tumor vascular integrity and metastasis. Cancer Lett..

[33] Sharma S, Goswami R, Zhang DX, Rahaman SO (2019). TRPV4 regulates matrix stiffness and TGFbeta1-induced epithelial-mesenchymal transition. J. Cell Mol. Med..

[34] Fang Y (2018). Pharmacological inhibition of TRPV4 channel suppresses malignant biological behavior of hepatocellular carcinoma via modulation of ERK signaling pathway. Biomed. Pharmacother..

[35] Tang B (2019). VPAC1 couples with TRPV4 channel to promote calcium-dependent gastric cancer progression via a novel autocrine mechanism. Oncogene.

[36] Yang, S. et al. Leukemia cells remodel marrow adipocytes via TRPV4-dependent lipolysis. *Haematologica*. **105**, 225763 (2019). 10.3324/haematol.2019.225763 PMC7604636 33131246

[37] Janssen DA (2011). The mechanoreceptor TRPV4 is localized in adherence junctions of the human bladder urothelium: a morphological study. J. Urol..

[38] Li Y (2016). Expression of a Diverse Array of Ca^2+^-Activated K+ Channels (SK1/3, IK1, BK) that Functionally Couple to the Mechanosensitive TRPV4 Channel in the Collecting Duct System of Kidney. PLoS ONE.

[39] Lin Y (2019). Serum calcium is a novel parameter to assess metabolic syndrome in endometrial carcinoma. J. Gynecol. Oncol..

[40] Grasset EM (2018). Matrix Stiffening and EGFR Cooperate to Promote the Collective Invasion of Cancer Cells. Cancer Res..

[41] Fujii S (2020). The TRPV4-AKT axis promotes oral squamous cell carcinoma cell proliferation via CaMKII activation. Lab. Invest..

[42] Yu S (2019). Transient receptor potential ion-channel subfamily V member 4: a potential target for cancer treatment. Cell Death Dis..

[43] Kadio B (2016). Calcium role in human carcinogenesis: a comprehensive analysis and critical review of literature. Cancer Metastasis Rev..

[44] Brenner W, Haber T, Junker K, Roos F, Thuroff JW (2015). [Bone metastasis by renal cell carcinoma. Importance of calcium and calcium-sensing receptor]. Urol. A.

[45] Wulaningsih, W. et al. Serum Calcium and the Risk of Breast Cancer: findings from the Swedish AMORIS Study and a Meta-Analysis of Prospective Studies. *Int. J. Mol. Sci*. **17**, 1487 (2016). 10.3390/ijms17091487 PMC5037765 27608013

[46] Wulaningsih W (2013). Serum calcium and risk of gastrointestinal cancer in the Swedish AMORIS study. BMC Public Health.

[47] Erratum. *Br. J. Pharmacol*. **174**, 116 (2017). 10.1111/bph.13672 PMC5341488 27933607

[48] Jones MC, Zha J, Humphries MJ (2019). Connections between the cell cycle, cell adhesion and the cytoskeleton. Philos. Trans. R. Soc. Lond. B Biol. Sci..

[49] Di Modugno F (2018). hMENA is a key regulator in endothelin-1/beta-arrestin1-induced invadopodial function and metastatic process. Proc. Natl Acad. Sci. USA.

[50] Ryskamp DA (2016). TRPV4 regulates calcium homeostasis, cytoskeletal remodeling, conventional outflow and intraocular pressure in the mammalian eye. Sci. Rep..

[51] Li X, Wang J (2020). Mechanical tumor microenvironment and transduction: cytoskeleton mediates cancer cell invasion and metastasis. Int. J. Biol. Sci..

[52] Amano M, Nakayama M, Kaibuchi K (2010). Rho-kinase/ROCK: A key regulator of the cytoskeleton and cell polarity. Cytoskeleton (Hoboken).

[53] Cheng Y, Shen P (2020). miR-335 Acts as a Tumor Suppressor and Enhances Ionizing Radiation-Induced Tumor Regression by Targeting ROCK1. Front Oncol..

[54] McCall PM, MacKintosh FC, Kovar DR, Gardel ML (2019). Cofilin drives rapid turnover and fluidization of entangled F-actin. Proc. Natl Acad. Sci. USA.

[55] Locard-Paulet M (2016). Phosphoproteomic analysis of interacting tumor and endothelial cells identifies regulatory mechanisms of transendothelial migration. Sci. Signal..

[56] Singh V (2017). Phosphorylation: Implications in Cancer. Protein J..

[57] Lee MH, Kundu JK, Chae JI, Shim JH (2019). Targeting ROCK/LIMK/cofilin signaling pathway in cancer. Arch. Pharm. Res..

[58] Lin T (2003). Rho-ROCK-LIMK-cofilin pathway regulates shear stress activation of sterol regulatory element binding proteins. Circ. Res..

