# Supplementary figures and images for: Calcium and TRPV4 promote metastasis by regulating cytoskeleton through the RhoA/ROCK1 pathway in endometrial cancer
Source: Cell Death Dis. 2020 Nov 23;11(11):1009. doi: 10.1038/s41419-020-03181-7 (PMC7683721; doi:10.1038/s41419-020-03181-7)

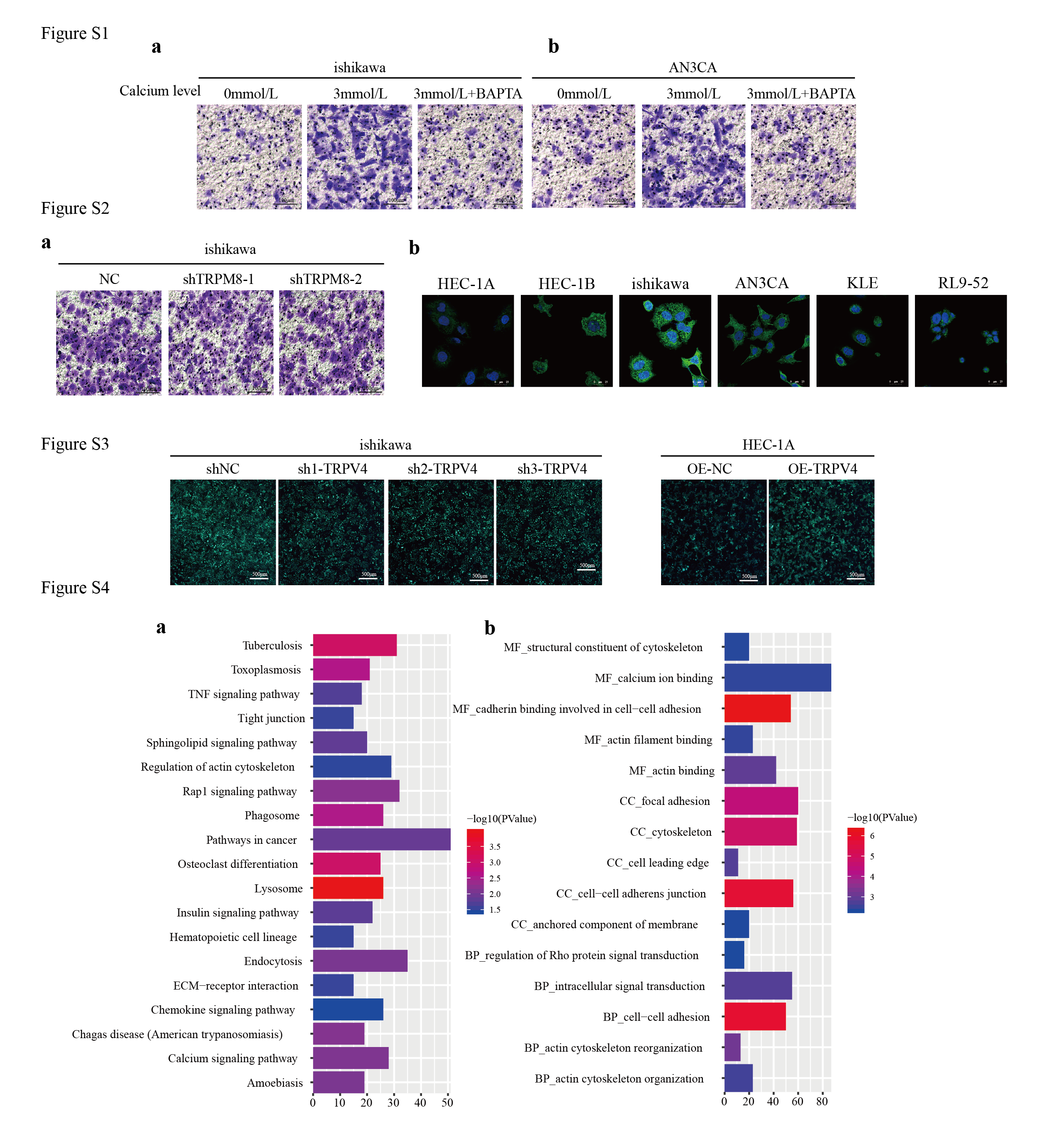

Supplement: Supplementary file 1 — Supplementary Figure S1-S4 [file 41419_2020_3181_MOESM1_ESM.png]

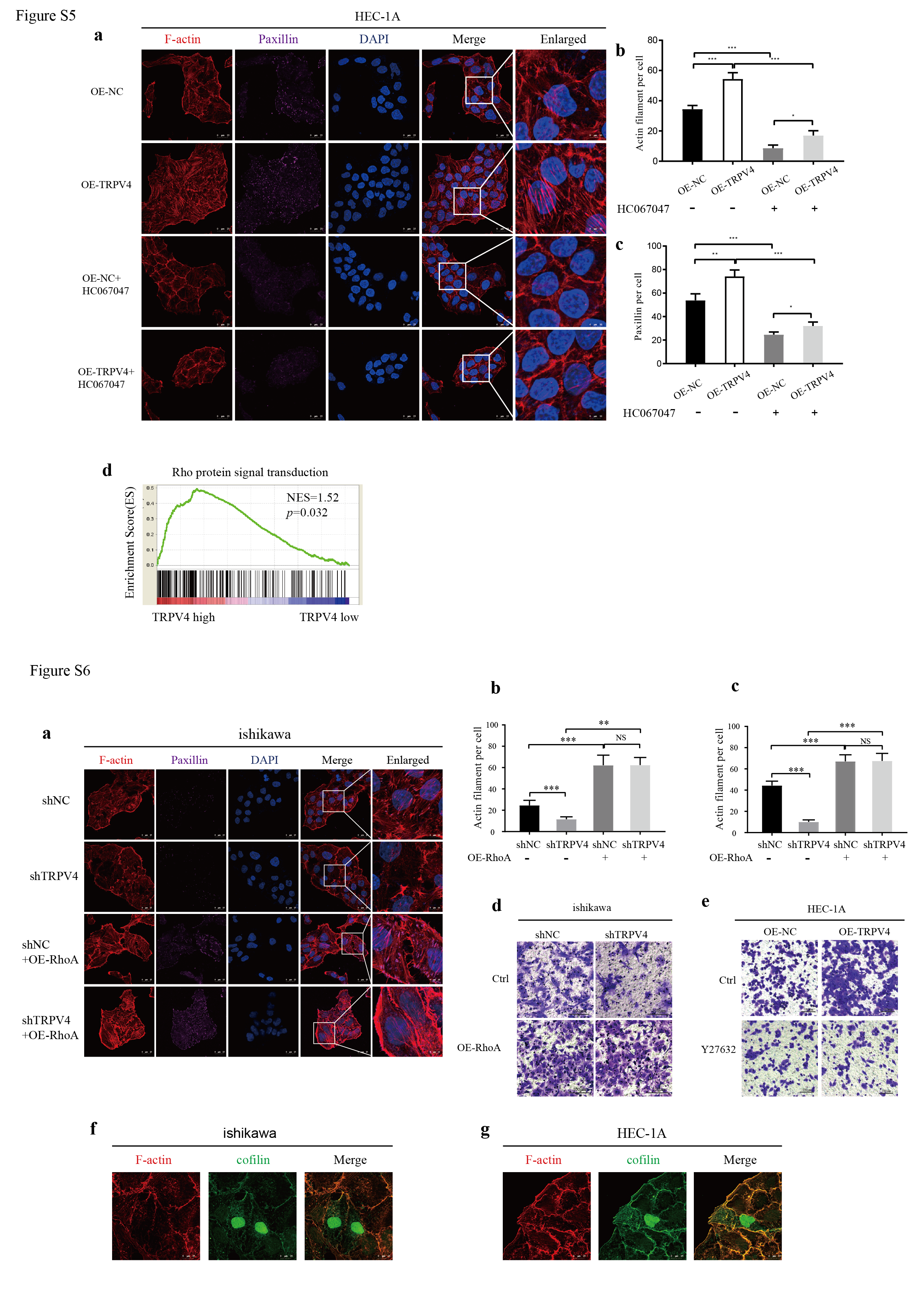

Supplement: Supplementary file 2 — Supplementary Figure S5-S6 [file 41419_2020_3181_MOESM2_ESM.png]
