# Supplementary material for: Calcium and TRPV4 promote metastasis by regulating cytoskeleton through the RhoA/ROCK1 pathway in endometrial cancer
Source: Cell Death Dis. 2020 Nov 23;11(11):1009. doi: 10.1038/s41419-020-03181-7 (PMC7683721; doi:10.1038/s41419-020-03181-7)
Supplement: Supplementary file 3 — Supplementary Figure Legends [file 41419_2020_3181_MOESM3_ESM.docx]

Supplementary Figure Legends

**Fig. S1. a**, **b.** The effects of calcium concentration and BAPTA-AM (10μM) on Ishikawa and AN3CA cell lines.

**Fig. S2.** **a**. metastatic capability of TRPM8 in ishikawa cell line. shTRPM8-1, knock down of TRPM8. **b**. Representative confocal images of expression of TRPV4 in 6 different cell lines by immunocytochemistry. Green indicates TRPV4 and blue indicates DAPI.

**Fig. S3.** Immunofluorescence showed the transfection efficiency. The plasmid is attached with GFP.

**Fig. S4.** **a**. GO and **b.** KEGG analysis of high and low expression of TRPV4 in TCGA dataset.

**Fig. S5.** Results of cytoskeleton assay of HEC-1A cells, visualized by confocal microscopy. Representative images are shown. Cell nuclei were stained with DAPI. Scale bar represents 25um or 10um. **b**, **c**. Statistical bar chart of the number of F-actin and PXN. **d** High expression of TRPV4 is enriched in the Rho protein pathway.

**Fig. S6.** **a**. Overexpression of RhoA increased the number of F-actin/paxillin in both shNC and sh-TRPV4 groups. Magnification 63×. Scale bar: 25 or 10 μm. **b**, **c**. Statistical numbers of actin filaments and PXN in each group. **d**. Migration in control and shTRPV4 conditions with RhoA antagonist in ishikawa cells. **e**. Migration in both OE-NC and OE-TRPV4 conditions with Y27632 (10μM) in HEC-1A cells. **f**, **g**. Co-localization of F-actin and confilin in ishikawa and HEC-1A cells.
